# Supplementary figures and images for: Interactions between Mycoplasma mycoides subsp. mycoides and bovine macrophages under physiological conditions
Source: PLoS One. 2024 Jun 27;19(6):e0305851. doi: 10.1371/journal.pone.0305851 (PMC11210856; doi:10.1371/journal.pone.0305851)

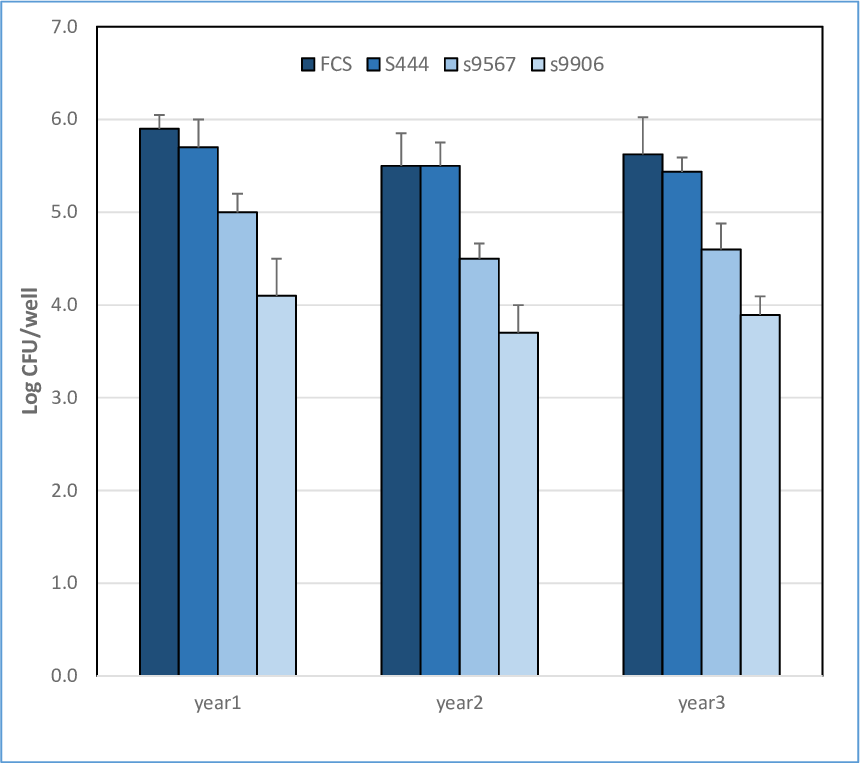

Supplement: S1 Fig — (TIF) [file pone.0305851.s001.tif]

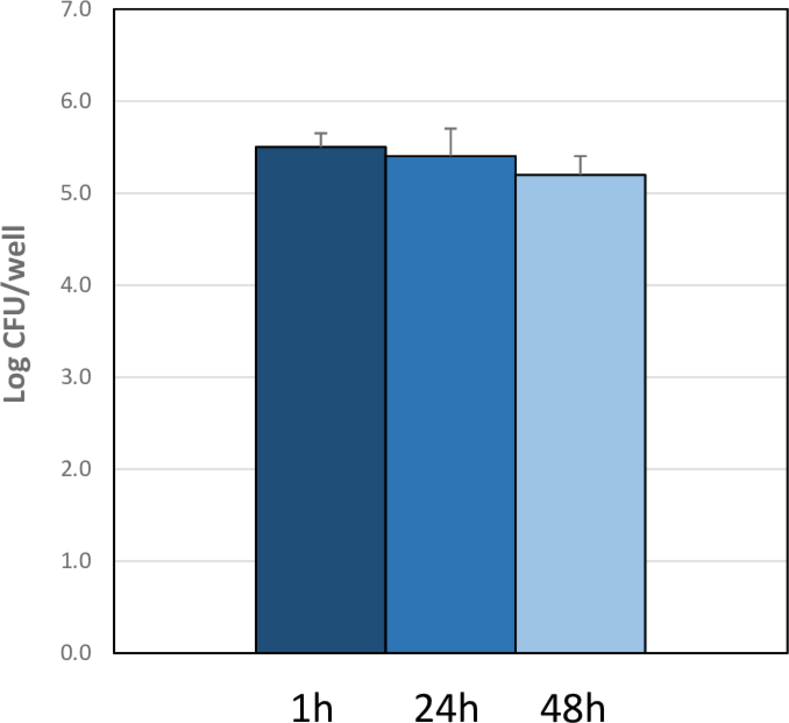

Supplement: S2 Fig — (TIF) [file pone.0305851.s002.tif]
