# Supplementary material for: Interactions between Mycoplasma mycoides subsp. mycoides and bovine macrophages under physiological conditions
Source: PLoS One. 2024 Jun 27;19(6):e0305851. doi: 10.1371/journal.pone.0305851 (PMC11210856; doi:10.1371/journal.pone.0305851)
Supplement: Attachment — Submitted filename: Comments to authors_PONE-D-23-32412.docx [file pone.0305851.s003.docx]

Comments to authors

In the manuscript by Totté et al., the authors have described interactions of Mycoplasma mycoides subsp. mycoides (Mmm) with bovine macrophages using in vitro killing and phagocytosis assays. While the manuscript focuses on an essential area of research of Contagious Bovine Pleuropneumonia by conducting experiments to identify interaction of macrophages (from the target species) with Mmm, the data reported in the manuscript is not enough to support the current conclusions. Moreover, the authors have made various highly speculative statements without providing any evidence whatsoever to in the manuscript.

Unfortunately, in its current form the manuscript cannot be accepted. The authors must provide further experimental data in order to consider the publication of the manuscript. These are mentioned below.

Line 108-109: In this manuscript, the methodology used to study the interaction of Mmm and macrophages is extremely important. So, Mmms were left with the macrophages for 1 hr in all the experiments? Were the cells washed before adding different serums.

The way methodology is written currently, it seems that both mycoplasmas and serums were added together. It is very important for the results observed in Fig 2 because if the mycoplasmas and serums were added together then its really not different compared to the data presented in Fig 1.

The authors need to clarify this.

Flow cytometry analysis (Line 130-132): The flow cytometry analyses are important in order to understand whether Mmms are internalised (gone inside of the macrophages) or associated (made complex and stuck outside) with macrophages. Based-on the current gating strategy how would you differentiate macrophage-associated Mmm from macrophage-internalised Mmm?

Given that Mmms are so small, it will be very challenging to differentiate the macrophage-associated Mmm from macrophage-internalised Mmm. Due to this limitation, both the externally bound Mmm (large proportion) and internalised (minimum proportion) to macrophages will generate the same double positive signal (FITC + PI). Therefore, to understand what is going on here the side scatter and forward scatter graphs must be presented. In addition, the gating strategy must be presented in the supplementary information.

Line 180: Based on the methodology, it doesn't see that the authors have focused on Mmm associated to the macrophages. Please clarify.

Line 181: The authors mention that "no cytopathological effects were detected on macrophages". Did the authors measure viability of the macrophages?

Line 186: This is bit confusing. MOI is always represented in relation to cells, but the authors are saying in the absence of macrophages. Please clarify. The data must be presented for MOI 1 and 100 even thought, these MOI showed a similar trend.

Line 205: How was this conclusion made? The authors just mentioned that that "overwhelming majority of Mmm survival at 24 h was due to extracellular mycoplasmas". I don't think that the authors can make this conclusion based on the current results. First, it needs to be demonstrated that Mmm can survive in macrophages. Given that very low number of Mmm were detected in the G+ treatment group, it is almost impossible to say that "complement had no effect on Mmm survival inside macrophages".

Line 212: Fig 3 Given that the authors performed experiments to assess intracellular survival of Mmm in macrophages, this should be macrophages-internalised and not macrophages-associated.

Line 220: These results are contradictory to the previous observations presented in Fig 3. In that, hardly any Mms were internalised but here the authors claim that over 90% were engulfed by the macrophages. Please clarify these contradicting results.

Also, proper gating strategy must be presented in the supplementary information including side and forward plots, gating for any possible clumps of neogreen mutant strain.

What was the rational of using 500-1000 MOI of Mmm in these experiments? In the previous Fig. the authors have mentioned that gentamicin did not kill Mmm at MOI higher than 100, indicating that Mmm higher than MOI of 100 are likely to associated with macrophages and NOT internalised. Please describe these discrepancies in your results.

Line 222: While it is understandable the rational of using neogreen expressing strain, I think there is bigger fundamental problem here. Given that no FITC signal was detected when MOI of 100 or less were used, it clearly indicates that both the WT and neogreen expressing strains are not behaving the same when it comes to their ability to infect or engulfed by macrophages. The first question, which must be answered that how comparable these two different strains are in terms of their ability to internalise or engulfed by macrophages. As far as I see, there is no data on this. Therefore, no conclusion can be made from any comparisons of the mutant and WT Mmm strains in this study.

Line 226: Given that the uptake (internalisation) is low, this conclusion cannot be made.

Line 242: Fig 4 By looking at the graphs, background fluorescence levels (hatched line) appear to be changing. Moreover, it should be at "zero" and not 10^2 or 10^3. It seems like that flow cytometry gating wasn't done properly and the gating strategy must be presented as the supplementary material.

Line 275: Mmm were diluted in PBS. Can the authors provide the source of PBS and other reagents used in the cell-culture experiments? Given that TNF levels can be easily induced even by a small amount of co-contaminating endotoxin levels in reagents, it is absolutely important to use certified endotoxin free reagents in cell culture experiments.

Line 278-279: I don't think, that is the correct way of reporting experimental findings. That's the variability of the outbred animals. You can't just take two best readings out of three. The results from the third animal must be reported. Otherwise, this experiment becomes, redundant.

Line 292: Fig 5 so, 2 animals each for serum 1 and serum 2? Is that correct?

Line 315: Yes, this is a most likely scenario, but authors kept saying that Mmms have internalised. So, results need to be updated.

Line 319: The authors should avoid using "internalisation or intracellular" because based on the results presented these conclusions can't be made.

The current flow cytometry analyses are not sufficient to conclude that Mmms are internalised into macrophages. Other methodology such as confocal microscopy could be used to analyse internalisation of Mmm into macrophages.

Line 333: Please see the comment for flow cytometry analysis.

Line 338: Again, please see the comment for line 220.

Line 345: But the authors just mentioned (in line 339) that the uptake of Mmm was independent of phagocytosis. Please clarify and discuss these in the discussion section.

Line 346: Again, the intensity can also increase if Mmm + macrophages form a complex and not necessarily internalised. So, the gating strategy must be presented with side and forward scatter graphs.

Line 352: Why did the authors not comment on the third animal, which showed substantial increase in TNF levels. The impact of that on the study should be discussed here.

Line 356: There are no evidence provided in the manuscript to support this. This statement is purely speculative. The authors should avoid making these types of statements without having any supportive data.

Line 362-368: All the above-mentioned issues must be addressed before making these i-iv conclusions. In it’s current form, the provided is not sufficient to make these conclusions.

Line 369-370: NO. This statement is totally speculative and should be rephrased.
