# Supplementary material for: Interactions between Mycoplasma mycoides subsp. mycoides and bovine macrophages under physiological conditions
Source: PLoS One. 2024 Jun 27;19(6):e0305851. doi: 10.1371/journal.pone.0305851 (PMC11210856; doi:10.1371/journal.pone.0305851)
Supplement: Attachment — Submitted filename: Manuscript Number PONE-D-23-32412 reviewers comments.docx [file pone.0305851.s004.docx]

**Manuscript Number PONE-D-23-32412**

Based on my review, I find the manuscript acceptable for publication. The authors have responded to the previous reviewer's comments well.

The knowledge generated from this study is crucial in enhancing our understanding of the Mmm interaction with macrophages.

Abstract:

Line 202: Write the abbreviation “MOI” in full

Introduction:

Line 36: Write WOAH in capital letters

Results:

Line 181: the sentence is not clear. It may be replaced with … “The mycoplasmacidal activity and inter-animal variability of bovine sera remained consistent throughout the study”.

Line 215: The efficacy of antibiotics against mycoplasmas is restricted to specific mycoplasma species and only reduces the concentration of mycoplasmas rather than killing the bacteria. Why was gentamycin used when there are more effective antibiotics like Tyrosin?

Discussion:

Line 336: It’s not clear. This information indicates that gentamicin was effective in preventing the survival of the bacteria within the macrophages, leading to the conclusion that the majority of the viable bacteria were located on the cell surfaces. Why was the gentamycin effective in this case?

Line 376: Use a uniform method of citation.
