# Supplementary material for: Interactions between Mycoplasma mycoides subsp. mycoides and bovine macrophages under physiological conditions
Source: PLoS One. 2024 Jun 27;19(6):e0305851. doi: 10.1371/journal.pone.0305851 (PMC11210856; doi:10.1371/journal.pone.0305851)
Supplement: Attachment — Submitted filename: ResponseToReviewersPONE-D-23-32412R2.docx [file pone.0305851.s005.docx]

**Answers to reviewer #3:**.

1/ Conclusions:

Considering the very interesting work done, I would recommend the authors to integrate the discussion commenting the results within the context of CBPP pathogenesis to increase the significance and the importance of the research. Find below some aspect to consider:
Use of MDMs. One of the main challenge in CBPP research is the difficulty of investigating the host-pathogen interaction at lung level in the early stages of disease onset. Due to the lack of laboratory animal models of CBPP infection, we urgently need simplified in vitro and ex vivo models to dissect the complex cattle immune response leading to disease pathology. Even if the ideal target cells to be investigated are the alveolar macrophages, harvesting of these cells from in vivo is not straightforward and require expert animal manipulation. The use of MDMs represent a valid alternative and it provided similar results with the advantage of starting from a simple blood sampling.

- *See added sentence lines 355-358 of the document with track changes.*

Complement. What is the current knowledge on the role of complement in the lung? May the complement contribute to down modulate the macrophage activation during the primary phase of infection and mycoplasma replication? *Complement used at non-bactericidal doses has no effect on TNF production by macrophages (Fig5, serum1).*

Or the abrogation of TNF-alpha production by MDMs observed in presence of bactericidal concentrations of complement is simply related to the reduction of Mmm concentration?

*- Yes.*

TNF-alpha. High concentrations of Mmm can induce TNF-alpha release by macrophages even in the absence of anti-Mmm antiserum. May this explain why there is no correlation between severe pathological findings and antibody response in CBPP infected animals?

*- Other reasons may be that antibody that are present locally in lungs differ from those detected in peripheral blood or that antibodies are involved in both pathology and protection.*

What is the role of this cytokine in cbpp pathogenesis and lung pneumonia? More importantly, what is the immune networking that is activated by tnf-alpha? Can the induction of TNF-alpha initiate the immune mechanisms that lead to CBPP fibrinous pneumonia?

*-Yes, given its role in exacerbated inflammation in other models (LPS-mediated sepsis, synovial rheumatoid) but other cytokines may be needed to attract phagocytes such as neutrophils that can release cytotoxic factors.*

Furthermore only high concentration of Mmm induced production of TNF-alpha. This indicates that while Mmm is replicating at low concentrations the immune system is not activated. May this explain why, in natural conditions, naïve animals requires multiple or prolonged exposure to diseased animal to get infected? Similarly, may this support the long incubation time sometimes observed in infected animals?

*- given the low level of phagocytosis by macrophages in the absence of antiserum, complement may be an important factor in the control of Mmm growth by infected animals as jstated lines 305-307.*

2/Other comments:
Minor editing corrections are required in the paragraph results Effect of bovine complement on the uptake of fluorescent Mmm by macrophages: line 232-257 (some errors: of of, MIF, FIg4c). *Done, see lines 231-239 of the document with track changes and Fig4’s legend.*
________________________________________

**Answer to reviewer #4:**

1/Abstract:
- Line 18-19: Write the abbreviation “MOI” in full. *Done.*

2/Introduction:
- Line 36: Write WOAH in capital letters. *Done.*

3/Results:
- Line 166-167: the sentence is not clear. It may be replaced with … “The mycoplasmacidal activity and inter-animal variability of bovine sera remained consistent throughout the study”. *Done*
- Line 215: The efficacy of antibiotics against mycoplasmas is restricted to specific mycoplasma species and only reduces the concentration of mycoplasmas rather than killing the bacteria. Why was gentamycin used when there are more effective antibiotics like Tyrosin?

*Because it does not penetrate inside cells at the concentration used in our study.*

3/Discussion:
- Line 334-336: It’s not clear. This information indicates that gentamicin was effective in preventing the survival of the bacteria within the macrophages, leading to the conclusion that the majority of the viable bacteria were located on the cell surfaces. Why was the gentamycin effective in this case?

*Gentamicin did not kill 100% of Mmm (i.e., in macrophages-free cutlures) at the high Mmm concentration used in flow cytometry. Therefore, it could not be used and assessment of fluorescent Mmm intracellular survival in macrophages could not be performed. The sentence has been reformulated for more clarity.*
- Line 341: Use a uniform method of citation. *Done*.
